# FPGA-Based Fused Smart-Sensor for Tool-Wear Area Quantitative Estimation in CNC Machine Inserts

**DOI:** 10.3390/s100403373

**Published:** 2010-04-07

**Authors:** Miguel Trejo-Hernandez, Roque Alfredo Osornio-Rios, Rene de Jesus Romero-Troncoso, Carlos Rodriguez-Donate, Aurelio Dominguez-Gonzalez, Gilberto Herrera-Ruiz

**Affiliations:** 1 HSPdigital–CA Mecatronica, Facultad de Ingenieria Campus San Juan del Rio, Universidad Autonoma de Queretaro, Rio Moctezuma 249, San Cayetano, C.P. 76807, San Juan del Rio, Qro., Mexico; E-Mails: mtrejo@hspdigital.org (M.T.-H.); troncoso@hspdigital.org (R.J.R.-T.); cdonate@hspdigital.org (C.R.-D.); auredgz@uaq.mx (A.D.-G.); 2 Facultad de Ingenieria, Universidad Autonoma de Queretaro, Cerro de las campanas s/n Col. Las Campanas, C.P. 76010, Queretaro, Qro., Mexico; E-Mail: gherrera@uaq.mx

**Keywords:** tool-wear area, vibration monitoring, current monitoring, smart-sensor, FPGA

## Abstract

Manufacturing processes are of great relevance nowadays, when there is a constant claim for better productivity with high quality at low cost. The contribution of this work is the development of a fused smart-sensor, based on FPGA to improve the online quantitative estimation of flank-wear area in CNC machine inserts from the information provided by two primary sensors: the monitoring current output of a servoamplifier, and a 3-axis accelerometer. Results from experimentation show that the fusion of both parameters makes it possible to obtain three times better accuracy when compared with the accuracy obtained from current and vibration signals, individually used.

## 1. Introduction

The manufacturing processes have been of great relevance in the economic development of many countries, and the constant claim for better productivity with high quality at low cost is a topic of great interest nowadays. These and other desirable requirements can be improved in the next generation of CNC (Computerized Numerical Control) machines, Mekid *et al*. [1]. Furthermore, the costs of the cutting tools and their replacement become an important amount of the total production costs (around 12%) according to Weckenmann *et al.* [2]. Therefore, several research works about the optimization of cutting conditions, detection and suppression of vibrations, detection and prevention of tool breakage and tool-wear state monitoring in chip-removing machining process have been made. The tool-wear can be classified into two main categories according to Kalpakjian and Schmid [3]: flank wear and crater wear. Flank wear is present in the incidence area of the tool and it is attributed to excessive rubbing with the machining surface at high temperatures. The crater wear is present just on the tool face and it is due to high temperatures between the tool and the chip, the chemical affinity of materials, and the excessive rubbing. Also, to carry out the tool-wear monitoring, two methods exist according to Liang *et al.* [4]: the direct method where vision systems and image processing are mainly utilized, implying an offline estimation; and the indirect method, more commonly utilized where the tool-wear state is qualitatively estimated from cutting forces, which are indirectly obtained through the use of some type of sensor such as accelerometers, dynamometers, acoustic emission sensors and current sensors, or the combined utilization of some of them (fused sensors).

Examples of developments for monitoring the tool-wear with one sensor are the works of Choudhury and Kishore [5] utilizing a dynamometer for sensing cutting forces, or Kopac and Sali [6] who make use of a microphone as sensor. Furthermore, in others investigations several sensors are utilized, such as Dimla and Lister [7] who utilize the cutting forces, measured through a dynamometer, and the vibrations obtained with an accelerometer to report a qualitative classification of the tool-wear state by means of neural networks. In the work of Cakan *et al.* [8] the behavior of tool-wear due to the insert coating is studied with TiN (titanium nitride) and CrN (chromium nitride) coated inserts, utilizing a dynamometer and a photo-electronic sensor that monitors the changes in the diameter of the piece in a turning process, and a qualitative comparison in the time domain of the signal behavior from both sensors is presented. On the other hand, Salgado and Alonso [9] employ a Hall-effect sensor, a dynamometer and a microphone to obtain current, force and acoustic emission signals, respectively, to quantitatively predict the flank wear in turning; Scheffer *et al.* [10] utilize multiple sensors including an acoustic emission sensor, a dynamometer, and an accelerometer, relating the acoustic emission signals and static force with the flank-wear for the quantitative prediction of tool-wear evolution in time, reporting a 5% error. The use of a fused sensor (acoustic emission and force), is also used by Deiab *et al.* [11] for the quantitative monitoring of tool-wear; polynomial classifiers and neural networks in the prediction are utilized obtaining an average accuracy of 92.04%. Kuljanic *et al.* [12,13] propose the vibration monitoring in a milling machine utilizing accelerometers and a dynamometer, then the signals are processed for extracting some statistical parameters. However, the processing is indirect and computed offline in a PC. A similar work is from Tarng and Chen [14], where neural networks and a dynamometer for chatter detection are utilized. From these woks, the importance of failure detection and tool-wear monitoring in cutting processes is evident, making of great relevance to count with a sensor or a fusion of sensors that are capable to acquire, process and show the result online. Though this problem has been widely studied and reported on literature, a sensor with embedded signal processing there has not been reported, that, based on primary sensors, determines the flank-wear area. Therefore, it is desirable to have a smart-sensor, defined as the one that gathers certain functionalities like processing, communication and integration, according to the classification given by Rivera *et al.* [15] and based on the definitions of the Institute of Electrical and Electronics Engineers (IEEE), that performs the desired characteristics specified by Mekid *et al.* [1], to quantitatively estimate the tool-wear state in inserts, being reliable and having the minimal error to improve its detection.

The new generation of manufacturing systems, according to Mekid *et al.* [1], should include some characteristics such as: integration, bidirectional stream of data, control loop process, predictive maintenance, and autonomous optimization. To facilitate these characteristics, the implementation of some functionality features like online monitoring of the machining process through reliable sensing techniques, is necessary.

This problem can be solved with the utilization of smart-sensors. Some examples of this type of sensors are the works of Hernandez *et al.* [16], where they utilize a Kalman filter to improve the response of several accelerometers employed in automobiles under performance tests; Rangel *et al.* [17] who developed a smart-sensor implemented in a field programmable gate array (FPGA) for jerk monitoring in CNC machines, utilizing a standard accelerometer as primary sensor and oversampling techniques to minimize the quantization noise; Granados *et al.* [18] accomplished the real-time high-resolution frequency measurement based on the implementation of the signal conditioner, analog-to-digital conversion, chirp z-transform, and spectral analysis to compose in this way the smart-sensor. Rivera *et al.* [19] present the auto-calibration and optimum response of an intelligent sensor with several nonlinear input signals through neural networks, achieving to introduce the system in a microcontroller unit applied to temperature monitoring. In other example, Jong *et al.* [20] handle the failure detection in an AC motor utilizing a smart-sensor with flux, Hall-effect sensors and accelerometers as primary sensors. The utilization of one or more primary sensors joined to hardware for processing, allows inferring the desirable parameter with higher accuracy, besides performing the task online.

The contribution of this work is the development of a fused smart-sensor in order to improve the online quantitative estimation of flank-wear area in CNC machine inserts, from the information provided by primary sensors such as the monitoring current output of a servoamplifier and an accelerometer. Additionally, this developed smart sensor adjusts the tool-wear area estimation considering the machining parameters of cutting depth and feed rate. Due to the fact that most investigations perform the signal processing from a primary sensor, or several of them and in a separated way, the proposed methodology compares results of tool-wear estimation from the feed-motor current signal, the vibration signal, and the fused signals in a turning process. They show that the estimation from the signal fusion minimizes the error on being compared against the estimation of a single sensor signal. It is the most utilized approach of previously reported works. To achieve this objective in the present work, a fused smart-sensor based on hardware signal processing (HSP) techniques capable of computing the tool-wear area estimation online, is developed thanks to the low-cost FPGA implementation of signal processing and conditioning.

## 2. Background

### 2.1. Tool-Wear

**Figure 1.**
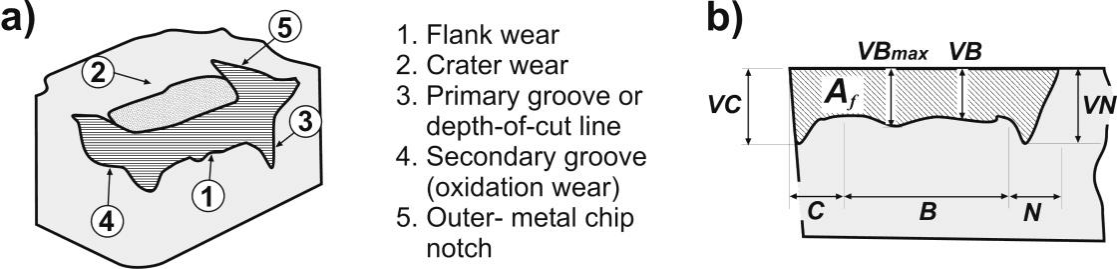
a) Types of tool-wear in carbide tools, b) Flank-wear area (*A_f_*), width of flank wear (*VB*) and *VB*_*ma*x_ in zone *B*, notch wear (*VN*) in zone *N*, and nose wear (*VC*) in zone *C*.

The tool-wear is a gradual process, where the worn rate depends on the workpiece and tool materials, the cutting fluids, and the cutting parameters, among others. Although, only flank wear and crater wear [3,21] are traditionally considered, there are also other kinds of tool-wear, *i.e.*, nose wear, oxidation wear, primary notch, *etc.*, as shown in Figure 1a [3].

The crater wear (Figure 1a, marked 2) appears at the attack face of the tool, and generally it is described as a diffusion mechanism caused by high temperatures in the tool-chip interface, apart from the chemical affinity between the workpiece and tool materials. Even when the crater wear affects the cutting process, this is determinant only under very high-speed conditions, and its measurement implies the utilization of a surface instrument to determine the maximum depth of the crater or the crater volume. In this way, most of low-speed industrial applications utilize the flank wear as main indicator in the tool change determination [21].

The flank wear (Figure 1a, marked 1) affects the tool incidence area and it is attributed to excessive rubbing with the machining surface at high temperatures [3]. In the same way, in Figure 1b, a lateral view of the tool-wear is presented, where the nose wear (*VC*) in region *C*, the width mean of flank wear, known as allowable wear land (*VB*) in region *B*, the width of maximum flank wear (*VB_max_*), and the notch wear (*VN*) in region *N* are shown, Boothroyd and Knight [21]. Most of reported investigations have been limited to the tool-wear estimation through the measurement of the central portion (*VB*) exclusively. However, this region represents a portion of the tool-wear only. As it is described in literature, other two kinds of tool-wear in the flank exist: the nose wear and the notch wear.

The measurement of flank-wear area *A_f_* (Figure 1b) gives an improved tool-wear estimator, as shown in the works of Scheffer *et al.* [10] and Sumit and Mingyuan [22]. The flank-wear area measurement has the advantage of considering the width of the flank wear, the nose wear and the notch wear, increasing the effectiveness on the estimation of tool-wear.

### 2.2. Tool-Wear Area Monitoring

Different research works have been focused on the tool-wear monitoring or failure detection, in a qualitative way. The current sensors (Hall-effect sensors) and vibration sensors (accelerometers) are commonly utilized. For instance, on the one hand, Li [23] has utilized the current signal as estimator employing a Hall-effect sensor to qualitatively monitor the tool-wear in a turning process, utilizing a prediction model. Li and Tso [24], monitor the current in a drilling process, applying signal processing and fuzzy techniques to estimate the tool-wear, also qualitatively. Reñones *et al.* [25] utilize the electric power consumption through current sensors, as well as the work of Romero *et al.* [26] where a qualitative classification of the tool-wear applied to failure detection is presented. In a similar approach, Franco *et al.* [27] utilize spindle driver current to monitoring the cutting tool condition in a drilling process. On the other hand, vibrations are utilized by Dimla and Lister [7] to make a qualitative classification of tool-wear by means of neural networks. Salgado and Alonso [28] monitor the vibration signals through an accelerometer to determine the flank wear with spectral singular analysis and neural networks. In these works, a single primary sensor is utilized, but the signal processing is performed in a computer, separately. For the present research, a fused sensor approach is proposed for improving the tool-wear estimation when compared against a single-sensor approach.

### 2.3. Fused-Sensor Approach

**Figure 2.**
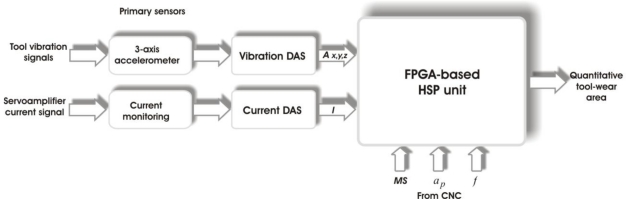
Block diagram of the proposed smart-sensor.

Figure 2 shows the block diagram of the proposed smart-sensor for tool-wear area monitoring. The vibration signals *A_x,y,z_*, are obtained from a standard 3-axial accelerometer and the feed-motor current *I* is directly taken from the current sensor at the servoamplifier. The smart sensor has two DAS (Data Acquisition System): one for acquiring vibrations and another one for the current acquisition. The signal processing is performed in the FPGA-based HSP unit. Besides, from the CNC machine, signals *MS* (Machining Start), depth of cut *a_p_* and feed rate *f* are inputs to the smart sensor. Finally, the quantitative estimation of the flank-wear area is supplied as the result.

## 3. Smart-Sensor

### 3.1. Proposed Methodology

**Figure 3.**
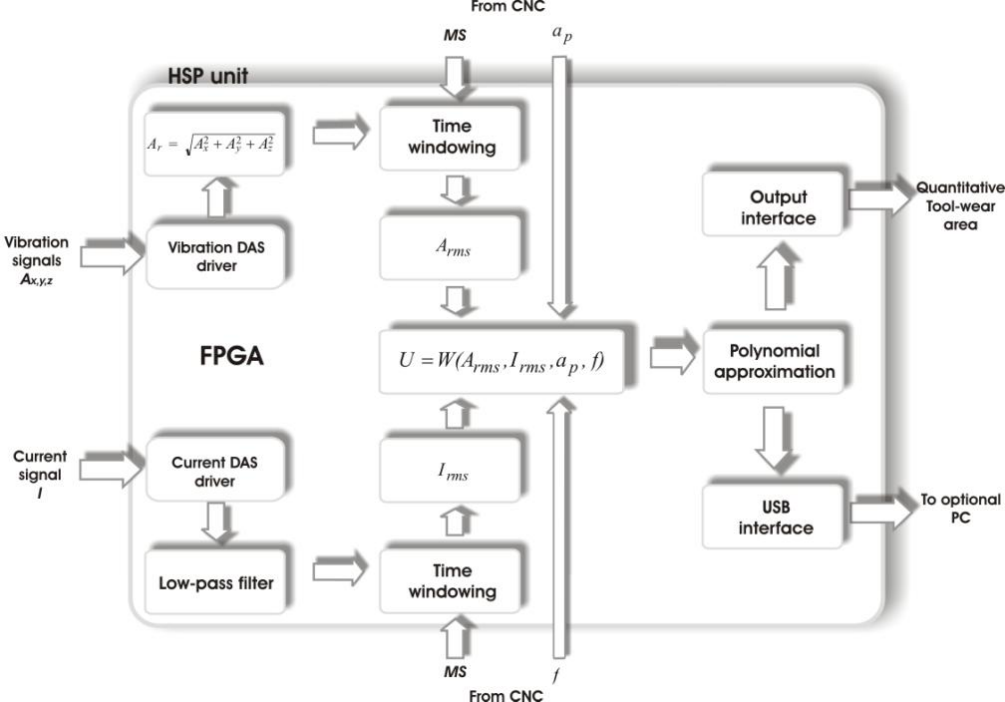
Block diagram of the FPGA-based HSP unit.

The block diagram of the HSP-unit internal structure to determinate the flank-wear area is shown in Figure 3. The vibration signals *A_x,y,z_* and the feed motor current *I*, are input signals in the vibration and current DAS driver, respectively. Then, the three vibration signals *A_x_*, *A_y_* and *A_z_*, acquired with the 3-axial accelerometer, are evaluated through Equation (1), obtaining in this way the resultant *A_r_* that contains the information from the sensed axes:

(1) $$A_{r}=\sqrt{A_{x}^{2}+A_{y}^{2}+A_{z}^{2}}$$

Then, this signal is time windowed for only taking into account the time when the cutting is made during the machining process. The time windowing initializes its processing when the CNC machine indicates it through a logical signal *MS* (Machining Start). Later, according to Equation (2), the RMS (Root-Mean Square) value of the produced signal by time windowing is obtained, being *A_ri_* the *i*-th sample and *m* the length of windowed samples. Simultaneously, the current signal is processed too, with an FIR low-pass filter (32^nd^ order and cutoff frequency *f_c_* = 120 Hz) and its corresponding time windowing. Afterwards, the *I_rms_* value of the current signal according to Equation (3) is obtained, where *I_i_* represents the *i*-th sample and *m* the length of windowed samples:

(2) $$A_{\mathrm{rms}}=\sqrt{\frac{\sum_{i=1}^{m}{\left(A_{\mathrm{ri}}\right)}^{2}}{m}}$$

(3) $$I_{\mathrm{rms}}=\sqrt{\frac{\sum_{i=1}^{m}{\left(I_{i}\right)}^{2}}{m}}$$

Starting from results in Equations (2) and (3), the fusion of this information is proposed as a simple weighting function [29] *U = W(A_rms_, I_rms_, a_p_, f)* that takes the effects of the machining parameters into account: depth of cut *a_p_*, and feed rate *f*. In a next step, the flank-wear area *A_f_* is obtained by a polynomial of approximation *A_f_(U)*. The polynomial approximation is shown in Equation (4), where *K_C_* is a scaling factor that depends on the depth of cut *a_p_*; *n* is the polynomial degree, coefficients *a_i_* and *K_C_* are fitted under Matlab from the experimental data. Finally, the obtained value is sent to the output interface for display, and optionally, results are sent to a personal computer through a USB interface for storage and further processing purposes:

(4) $$A_{f}\;(U)=K_{C}\sum_{i=0}^{n}a_{i}U^{i}$$

### 3.2. Signal Processing

**Figure 4.**
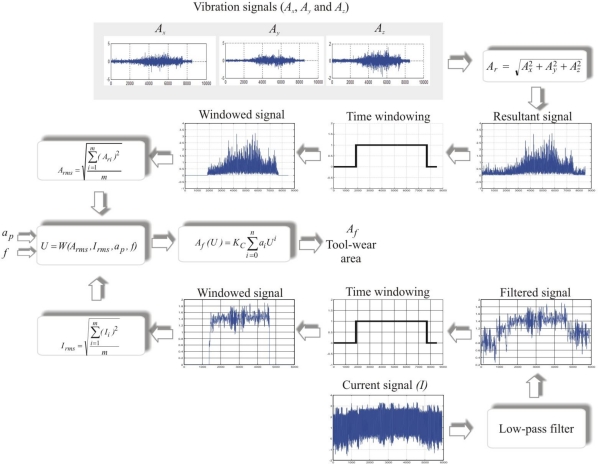
Vibration and current signal processing.

Figure 4 shows the vibration and current signals processing flow up to the evaluation in the polynomial approximation for obtaining the quantitative estimation of the flank-wear area *A_f_*. On the one hand, the processing flow starts with the vibration signal acquisition; then, it computes the vibration resultant, and after a time windowing the RMS value *A_rms_* is obtained. On the other hand, the current signal is acquired and filtered, then a time windowing is applied to calculate the RMS value *I_rms_* later on. The next step is the signal fusion through the weighting function that combines acceleration, current, and machining parameters. Finally, the tool-wear area is estimated with a polynomial approximation.

## 4. Experimental Setup

**Figure 5.**
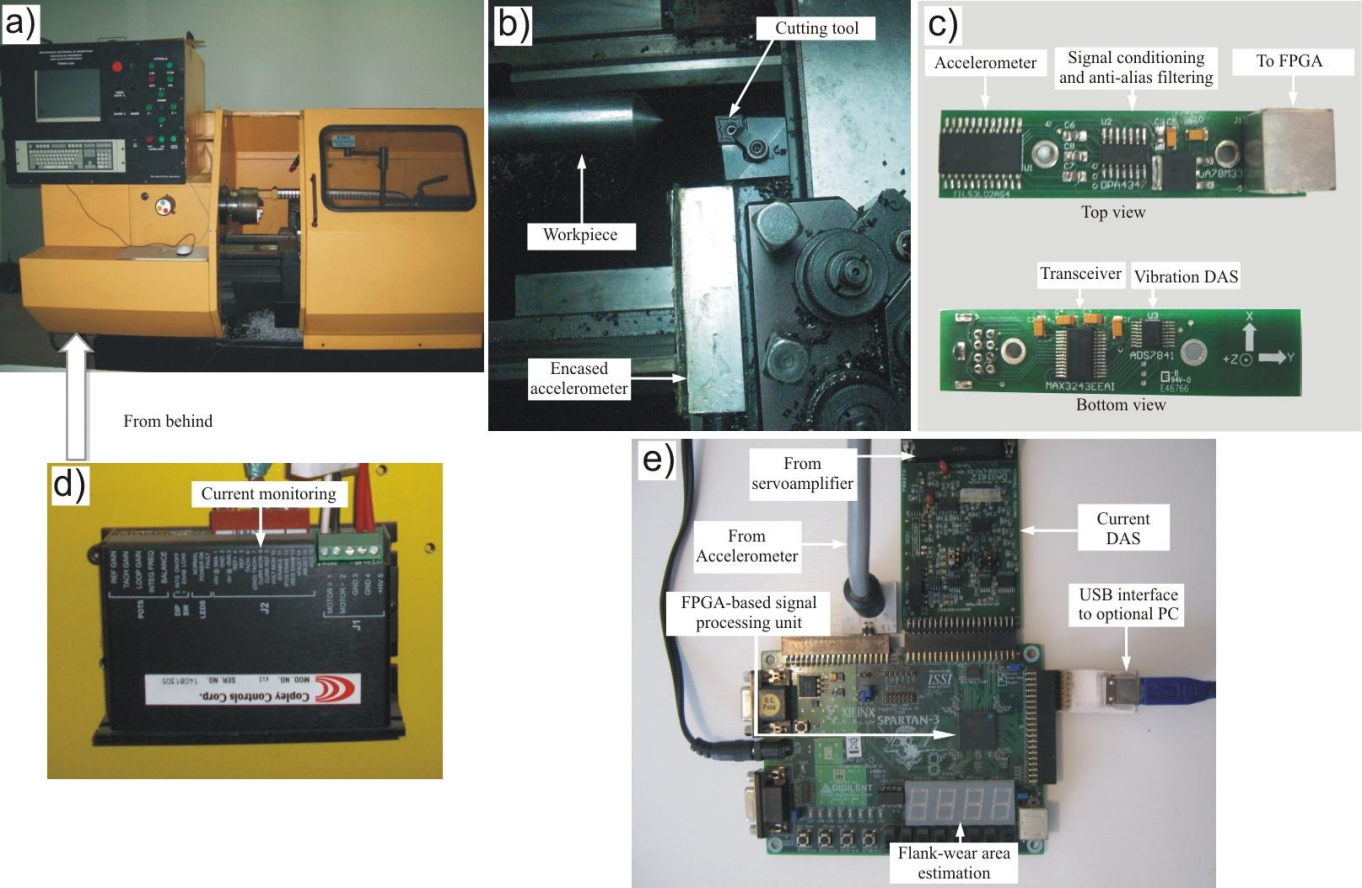
Experimental setup. (a) Retrofitted to CNC lathe. (b) Encased accelerometer. (c) Top and bottom view of the accelerometer board. (d) Servoamplifier. (e) FPGA-based signal processing unit.

The experiments were done in the retrofitted to CNC lathe shown in Figure 5a. The encased accelerometer, the cutting tool (insert) and workpiece can be seen in Figure 5b. Figure 5c depicts the top and bottom view of the accelerometer board, which includes signal conditioning and the vibration DAS. Likewise, the servoamplifier is shown in Figure 5d, and Figure 5e shows the FPGA-based signal processing unit utilized in the experiments, along with the current DAS.

In order to acquire the vibration signals of the machining process, the accelerometer board shown in Figure 5c is composed by a 3-axial LIS3L02S4 accelerometer from STMicroelectronics with an acceleration range of ± 2 g (±19.62 m/s^2^), mounted in the board with the signal conditioning and anti-alias filtering which is encased in aluminum and placed near the cutting tool. This accelerometer board also contains the vibration DAS consisting on a 4-channel, 12-bit sampling ADC with a sampling rate *f_s_* = 1,500 Hz that provides the digitized vibration signals, and the communication between the instrumentation system and the FPGA unit is performed with a MAX3243 transceiver.

The current signal is obtained from a typical servoamplifier, from Copley Controls Corp model 413 Series Tachometer DC-Brush, configured in current mode as shown in Figure 5d. The quantization process of the current signal is done in the current DAS board (Figure 5e). This board has two analog input channels from an ADC at 50 kSPS (kilo Samples Per Second), two analog output channels from a DAC at 100 kSPS, and eight digital inputs.

Finally, also shown in Figure 5e, to obtain the quantitative flank-wear area monitoring, the proposed HSP processing unit of the fused smart-sensor is implemented into a 200,000-gate Xilinx Spartan-3 FPGA, where the result of flank-wear area estimation is shown online in a 4-digit 7-segment LED display. The result is also available via the USB interface to an optional PC, for storage and further processing purposes.

### 4.1. Weighting Function Parameters

**Figure 6.**
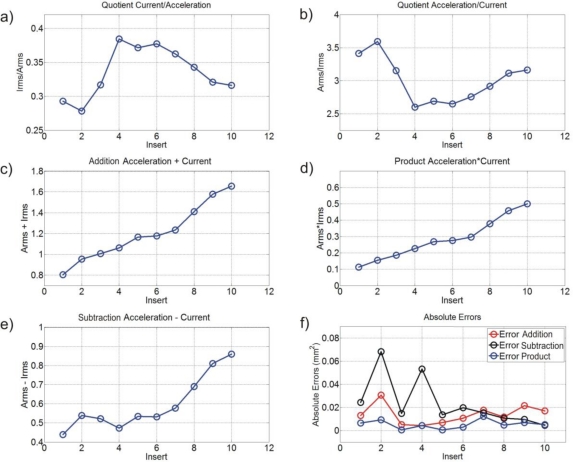
Exploration of weighting function parameters.

In order to find a simple function that improves the flank-wear area estimation, the exploration of weighting functions through addition, subtraction, product and quotient operations between *A_rms_* and *I_rms_* parameters, is made. Ten experiments with ten different inserts are utilized for this purpose: the results are shown in Figure 6. In Figures 6a and 6b, the behavior of *I_rms_/A_rms_* and *A_rms_/I_rms_* are not monotonic, for this reason, these operations are not considered. Figures 6c, 6d and 6e, show better responses; then, these data are processed to obtain a polynomial approximation under Matlab, and the corresponding absolute errors are calculated as depicted in Figure 6f. The weighting function that relates *A_rms_* and *I_rms_* parameters with product shows lower error. Then the weighting function is proposed to contain the product of current and vibration, to further include the machining parameters.

### 4.2. Machining Parameters in the Weighting Function

The weighting function must include adjusting factors that consider the effects of the machining conditions. Equation (5) is proposed as the full weighting function, including the product of current and vibrations, alongside two adjusting factors; a scaling factor *K_S_* and a shifting factor *K_D_*. By considering these adjusting factors, the smart sensor is insensitive to changes in cutting conditions:

(5) $$U=K_{S}\;A_{\mathrm{rms}}I_{\mathrm{rms}}+K_{D}$$

In order to fit the weighting function with the adjusting factors, several tests were performed. These tests consist on ten turning cycles of ten inserts with different wear area for each of five depths of cut *a_p_* and five feed rates *f* as summarized in Table 1. The cutting tools are coated carbide inserts CNMG433MA machining a conic surface because in this way the three components of acceleration can be analyzed (oblique cutting) over at medium-carbon steel (AISI 1,045), stock material diameter *D* = 25.4 *mm*, without coolant liquid. The inserts were classified according to their degree of tool-wear, in a range that goes from new, medium tool-wear to broken inserts. Then, their flank-wear area was estimated for calibration purposes using a NIKON EPIPHOT 200 microscope and further image processing techniques. The inserts were provided by a local metal-mechanic industry with the aim of having a representative sample of an industrial process. The cutting conditions were selected according to the general recommendations for turning operations of medium and high-C steels with coated carbide tools, as suggested in Kalpakjian and Smith [3]. The capabilities of the lathe were also considered.

## 5. Results and Discussion

This section presents the tool-wear area estimation utilizing three approaches: the first methodology makes the estimation using the current signal only, the second methodology is based on the vibration signal, and the third one is the proposed fused method. Results from these methodologies are compared to demonstrate the improvement of the proposed smart sensor. The three approaches use current and vibration signals that were simultaneously acquired for each experiment thanks to the FPGA reconfigurability for implementing the HSP unit, without changing the hardware configuration.

### 5.1. Current-Based Estimation

**Figure 7.**
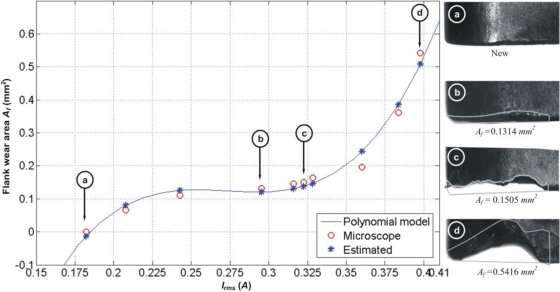
Flank-wear area estimation based on current signal, showing the micrograph of selected inserts and their corresponding tool-wear area.

The current signals are processed with the polynomial fitting methodology presented by Scheffer *et al.* [10], for the machining parameters specified in Experiment 1 from Table 1. The flank-wear area *A_f_* as a function of current signal *I_rms_* is estimated and then compared against the measured flank-wear area as shown in Figure 7. This figure also shows the cutting tool micrograph and its quantitative flank-wear areas for selected inserts (marked a, b, c and d). The 3^rd^ degree polynomial coefficients of *A_f_(I_rms_)* are fitted under Matlab from the experimental results to obtain (6). The absolute error for this estimation reports a mean of *μ* = 0.0207 *mm^2^* and a standard deviation of *σ* = 0.0118 *mm^2^*. The estimated relative error for this approach is 12.6%.

(6) $$A_{f}\;(I_{\mathrm{rms}})=213.8785\;I_{\mathrm{rms}}^{3}-175.1362\;I_{\mathrm{rms}}^{2}+47.5507\;I_{\mathrm{rms}}-4.1563$$

The polynomial fitting shown in Equation (6) is valid for the specified machining parameters. If machining conditions, such as feed rate or depth of cut are changed, to recalculating the fitting coefficients is necessary.

### 5.2. Vibration-Based Estimation

**Figure 8.**
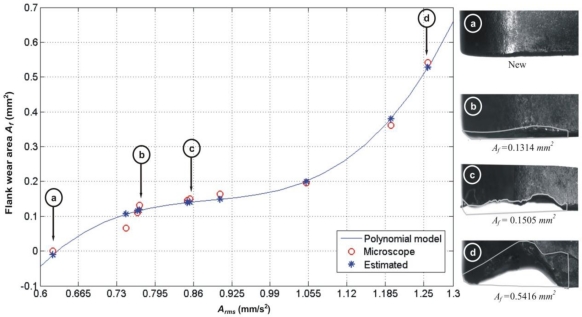
Flank-wear area estimation based on vibration signals.

This experiment utilizes the RMS value of vibration signals *A_rms_* for the estimation with the polynomial fitting methodology presented by Scheffer *et al.* [10] for the machining parameters specified in Experiment 1 from Table 1. In Figure 8, results for this approach as estimator of flank-wear area can be seen. In the same way that the RMS values of current signal in order to obtain the equation of flank-wear area *A_f_* as function of *A_rms_* values, a polynomial of 3^rd^ degree was fitted under Matlab. The fitted polynomial of *A_f_(A_rms_)* is shown in Equation (7). In this case, the mean of the absolute error from the estimation is *μ* = 0.0139 *mm^2^*, with a standard deviation of *σ =* 0.0105 *mm^2^* and a relative error of 11.99%.

(7) $$A_{f}\;(A_{\mathrm{rms}})=6.3008\;A_{\mathrm{rms}}^{3}-16.7350\;A_{\mathrm{rms}}^{2}+14.9711\;A_{\mathrm{rms}}-4.3642$$

Similar to the current approach, if machining conditions are changed in vibration monitoring, it is necessary to recalculate the fitting coefficients of the polynomial.

### 5.3. Fused Current-Vibration Estimation

The fused smart-sensor proposed methodology in this work consists on the fusion of current, vibration and machining parameters as stated in Equation (5). Equation (8) shows the resultant weighting function from experimental data, considering the whole set of machining conditions stated in Table 1, where *K_S_* = 1.3860 and *K_D_* = 0.5836. A 3^rd^ degree polynomial approximation under Matlab is fitted to obtain Equation (9) from the weighting function:

(8) $$U=1.3860\;A_{\mathrm{rms}}I_{\mathrm{rms}}+0.5836$$

(9) $$A_{f}\;(U)=30.1716\;U^{3}-24.8065\;U^{2}+6.9746\;U-0.5220$$

**Figure 9.**
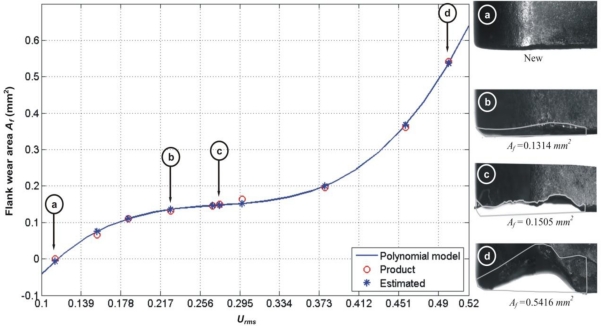
Flank-wear area estimation based on the fused smart-sensor methodology.

In order to make a fair comparison between the proposed fused methodology results against the reported single-parameter approaches, the flank-wear area is estimated with the same machining parameters from Experiment 1 described in Table 1. Figure 9 shows the tool-wear area estimation with the smart sensor methodology. The relative error for the fused approach is 3.7% with a mean *μ =* 0.0053 *mm^2^*, and a standard deviation of *σ =* 0.0036 *mm^2^*.

Table 2 summarizes results from the three methodologies under the same machining conditions as stated in Experiment 1 from Table 1.

The fitted polynomial in the proposed smart sensor methodology is capable of changing the machining parameters without requiring the coefficient recalculation different from the single parameter approaches that are fitted for a single machining condition. In order to demonstrate this feature, Table 3 shows the tool-wear area estimation errors for the whole set of experiments described in Table 1.

### 5.4. Discussion

The fused smart-sensor methodology developed in this work improves the tool-wear area estimation, achieving less than four percent of relative error when compared against over ten percent obtained with single-parameter estimations. This comparison was performed considering the same machining conditions for the three approaches. The single-parameter approaches are limited to a single machining condition for their tool-wear area estimation, whereas the fused methodology is not limited because the weighting function includes the effects of machining parameters.

When machining conditions are changed in the proposed smart sensor, the weighting function adjusts the tool-wear area estimation to consider these conditions. Results from Table 3 show that for five different feed rates and five different depths of cut the behavior of the estimation has repeatability, provided by the mean and the standard deviation of the absolute error. Additionally, the relative error on the tool-wear area estimation for the set of experiments is kept below 10%, average.

## 6. Conclusions

The present investigation develops a fused smart-sensor to quantitatively estimate the flank-wear area in inserts for machining processes. The main contribution of this work is to improve the estimation of the tool-wear state by combining current and vibration signals in a fused approach and by including the effects of different machining conditions. In order to diminish the error in the flank-wear area estimation a simple weighting function is proposed based on the product of the current and vibration signals alongside the machining parameters. Experimentation shows that the proposed parameter fusion improves results when compared against single-parameter approaches. Results for changing machining conditions show repeatability in the smart sensor methodology. The low-cost FPGA implementation of the HSP unit makes the system capable of performing an online processing. The application of the fused smart-sensor developed in this work can be suitable for other machining processes in the new generation of manufacturing machines for further investigations.

## Figures and Tables

**Table 1.** Cutting conditions for experimentation.

**Experiment**	**Feed rate *f* (*mm/rev*)**	**Depth of cut *a_p_* (*mm*)**	**Cutting speed *v_c_* (*m/min*)**	**Tool**	**Inserts with different tool-wear degree**
1	0.3333	1.5	72	Coated Carbide CNMG433MA Medium Cutting	10 inserts from new, medium tool-wear to broken
2	0.2778				
3	0.2222				
4	0.1667				
5	0.1111				
6	0.3333	2.5			
7		2.0			
8		1.5			
9		1.0			
10		0.5			

**Table 2.** Absolute errors of single- and fused-parameter estimation.

**Current based-estimation error (mm^2^)**		**Vibration based-estimation error (mm^2^)**		**Current and Vibration based estimation error (mm^2^)**	
**Mean (μ)**	**Standard deviation (σ)**	**Mean (μ)**	**Standard deviation (σ)**	**Mean (μ)**	**Standard deviation (σ)**
0.0207	0.0118	0.0139	0.0105	0.0053	0.0036

**Table 3.** Smart sensor tool-wear area estimation errors for different machining experiments.

**Experiment**	**Feed rate *f* (*mm/rev*)**	**Depth of cut***a_p_***(*mm*)**	**Cutting speed *v_c_* (*m/min*)**	**Current and Vibration based estimation absolute error (mm^2^)**	
				**Mean (μ)**	**Standard deviation (σ)**
1	0.3333	1.5	72	0.0053	0.0036
2	0.2778			0.0396	0.0533
3	0.2222			0.0599	0.0503
4	0.1667			0.0671	0.0631
5	0.1111			0.0650	0.0796
6	0.3333	2.5		0.0719	0.0504
7		2.0		0.0228	0.0255
8		1.5		0.0053	0.0036
9		1.0		0.0575	0.0663
10		0.5		0.0701	0.0902
